# Short and long term axotomy-induced ERG changes in albino and pigmented rats

**Published:** 2009-11-17

**Authors:** Luis Alarcón-Martínez, Pedro de la Villa, Marcelino Avilés-Trigueros, Román Blanco, Maria P. Villegas-Pérez, Manuel Vidal-Sanz

**Affiliations:** 1 Departamento de Oftalmología, Facultad de Medicina. Universidad de Murcia. 30100 Murcia, Spain; 2 Departamento de Fisiología, Facultad de Medicina. Universidad de Alcalá. 28871 Alcalá de Henares, Spain; 3 Departamento de Cirugía, Facultad de Medicina. Universidad de Alcalá. 28871 Alcalá de Henares, Spain

## Abstract

**Purpose:**

To investigate the different components of full-field flash electroretinogram (ERG) responses in adult albino and pigmented rats at various time intervals following optic nerve transection (ONT).

**Methods:**

In adult Sprague-Dawley (SD, albino) and Piebald-Viral-Glaxo (PVG, pigmented) rats, the left optic nerve was transected intraorbitally to induce retinal ganglion cell (RGC) death. ERG responses were recorded simultaneously from both eyes beforehand and at 1, 2, 4, and 12 week intervals after ONT. The ERG a- and b-waves and the scotopic threshold responses (STR) were analyzed in scotopic conditions. White light stimuli of intensities ranging from 10^−6^ to 10^−4^ cd·s·m^−2^ were used to record the positive and negative scotopic threshold responses (pSTR and nSTR), while stimulus light intensities ranging from 10^−4^ to 10^2^ cd·s·m^−2^ were used to analyze the a- and b-wave amplitudes of standard ERG recordings.

**Results:**

In the albino rats, one week after intraorbital ONT, the STR mean amplitude decreased significantly, to approximately 60% of the values registered for the contralateral eye (p<0.05), which had not been operated on; standard ERG a- and b-waves showed a small reduction in amplitude—to approximately 85%. By two weeks after ONT, the STR mean amplitude was approximately 40% that of the contralateral eye, while the a- and b-wave amplitudes had further decreased to approximately 75%. Four weeks after ONT, the STR had fallen to 60% of that of the contralateral eyes, whereas the a- and b-waves reached values of approximately 90%. Twelve weeks after ONT, the STR remained significantly reduced to approximately 45%, whereas the a- and b-waves reached values of approximately 90%. In the pigmented rats, one week after intraorbital ONT, the mean amplitude had decreased significantly, to approximately 60% for the pSTR and to 80% for the nSTR of the values registered for the intact contralateral eye (p<0.05); while the standard ERG a- and b-waves showed a small reduction in amplitude to approximately 90%. Two weeks after ONT, the STR mean amplitude was approximately 55%, while the a- and b-wave amplitudes had further decreased to approximately 65%. Four weeks after ONT, the STR also was significantly reduced, to only 40%, whereas the a- and b-waves reached values of approximately 60%. Twelve weeks after ONT, the pSTR and nSTR remained significantly reduced to approximately 40% and 70%, respectively; whereas the a- and b-waves reached values of approximately 80%.

**Conclusions:**

Optic nerve injury results in transient reductions of the major ERG components, the a- and b-waves, as well as permanent reductions of the early components of the ERG, the negative and positive scotopic threshold responses. Because ONT induces massive RGC loss, it is likely that permanent reduction in the STR represents a lack of the RGC population, thus highlighting the importance of the STR recordings as an electrophysiological tool for the assessment of RGC function.

## Introduction

An electroretinogram (ERG) measures full-field retinal potential from the cornea and it is commonly used in retinal function studies. Major components of the ERG are related to specific retinal cell populations. Under scotopic conditions, the initial negative wave recorded after a bright full-field stimulus (a-wave) is generated by photoreceptor phototransduction; while the b-wave, the prominent positive wave that follows the a-wave, is mainly generated by depolarization of ON-bipolar cells and Müller cells [1,2]. In addition to the a- and b-waves, oscillatory potentials (OPs) are another main component of the ERG. OPs appear superimposed on the b-wave and are thought to arise from feedback circuitries as well as from amacrine cells [3].

Unlike these relations between ERG waves and retinal cell populations, other neurons (i.e., retinal ganglion cells, RGC) have not yet been definitively linked to any electrical component of the ERG. Exposure to very dim light flash stimuli in the dark-adapted retina induces a small ERG response with a positive and negative component, with specific implicit times; these two waves are known as positive and negative scotopic threshold responses (pSTR and nSTR), respectively, and apparently have their origin in the innermost retina [4–6], where the RGC bodies are located. ERG studies performed in cats [4], pigmented rats [7], pigmented mice [8,9], monkeys [10], and humans [11] indicate that the RGC population is responsible at least in part for the generation of the pSTR and nSTR responses, although these STR responses may also contain a contribution of amacrine cells [8,9,11].

Compared to pigmented rats, albino rats have poor vision [12–14], which may be attributable to certain features of the visual system of the albino rat, such as a smaller amount of pigment in their ocular tissues, a smaller number of photoreceptors [15], or a smaller ipsilateral retinal projection [16,17]. Nevertheless, the albino rat has been the animal of choice for many experimental models involving RGC injury, including transient ischemia of the retina induced by elevation of the intraocular pressure [18] or by selective ligature of the ophthalmic vessels [19–24]; ocular hypertension induced by laser photocoagulation of the limbar tissues [25,26]; and optic nerve axotomy by complete crush [27] or transection [28–30]. A classic model to study injury-induced RGC death involves optic nerve lesions in the albino rat. Indeed, detailed quantitative studies on the effects of the distance at which axotomy is performed on the time course and amount of RGC loss [27,28,30–32] have shown that optic nerve transection (ONT) close to the eye induces two phases of RGC death [28,31]: an initial rapid phase involving the loss of approximately 80% of the RGC population between day 4–5 and day 14 after injury [28,31,33]; and a second, more protracted phase involving the loss of 15%–20% of the remaining RGCs and that extends for over a year after ONT [28]. Although this type of injury to the visual system of the adult albino rat results in several alterations in their functional [34–37] and metabolic [38,39] properties, as well as in the regulation of a substantial number of genes [30,40,41], the long-term changes in the ERG relating to albino rat retina have not been studied in detail.

It is commonly accepted that ONT results in the loss of RGCs, but not of other retinal neurons [28,42]. Our experimental design appears suitable to investigate the ERG components that may be altered as a consequence of the loss of this neuronal population. Therefore, for the present study, we have extended our previous work on axotomy-induced RGC death and investigated the different components of the full-field flash ERG in the adult albino rat at various time intervals after ONT. In addition, and to enable comparison with previous studies [7,43,44], we have also studied these responses in the adult pigmented rat. We found that optic nerve (ON) injury results in transient reductions of the major ERG components, the a- and b-waves, as well as in permanent reductions of the early components of the ERG—the negative and positive scotopic threshold (STR) responses. Overall, our data are in agreement with previous studies of the pigmented rat [7,44] and provides new and original information regarding the adult albino rat.

## Methods

### Animals

Female adult albino Sprague-Dawley (SD; 180–220 g) and pigmented (Piebald-Viral-Glaxo, PVG; 180–220 g) rats were treated according to institutional guidelines: the European Union regulations for the use of animals in research; the ARVO statement for the use of animals in ophthalmic and vision research; and to the guidelines published by the Institute for Laboratory Animal Research (Guide for the Care and Use of Laboratory Animals). Rats, kept in a 12 h light-dark cycle, were anaesthetized with an intraperitoneal (I.P.) injection of a mixture of ketamine (70 mg/kg Ketalar®, Pfizer, Alcobendas, Madrid, Spain) and xylazine (10 mg/kg Rompun®, Bayer, Kiel, Germany) in 0.1 ml saline. Four groups of albino rats, groups I (n=6), II (n=7), III (n=9), and IV (n=9) were processed at 1, 2, 4, and 12 weeks after ONT, respectively; while four groups of pigmented rats, groups V (n=8), VI (n=5), VII (n=6), and VIII (n=5) were also processed at 1, 2, 4, and 12 weeks after ONT, respectively.

### Optic nerve transection

The left ON was sectioned close to its origin in the optic disc, following the protocols mentioned above that are standard in our Laboratory [21,22,26,27,29,45–49]. In brief, to access the ON at the back of the eye, an incision was made in the skin overlying the superior orbital rim, the supero-external orbital contents were dissected, and the superior and external rectus muscles were sectioned. The dura mater of the ON was opened longitudinally, and the ON was completely transected as close to the eye as possible. Care was taken not to damage the retinal blood supply, which enters the eye separately in the inferonasal aspect of the ON sheath [50,51].

### Electroretinography

The animals were dark adapted overnight before the ERG recordings, and their manipulation was done under dim red light (λ>600 nm). The rats were anaesthetized and bilateral pupil mydriasis was induced by applying a topical drop of 1% tropicamide (Colircusi tropicamida 1%®; Alcon-Cusí, S.A., El Masnou, Barcelona, Spain) to both eyes. The light stimulation device used was a Ganzfeld dome, which ensures a homogeneous illumination anywhere in the retina, with multiple reflections of the light generated by light emitting diodes (LED), which provided a wide range of light intensities. For high intensity illuminations, a single LED placed close (1 mm) to the eye was used. Light intensity was calibrated by a dual-biosignal generator device specifically adapted for ERG responses. The recording system comprised Burian-Allen bipolar electrodes (Hansen Labs, Coralville, IA) with a corneal contact shape; a drop of methylcellulose, 2% (Methocel 2%®; Novartis Laboratories CIBA Vision, Annonay, France) was placed between the eye and the electrode to maximize conductivity of the generated response. The reference electrode was placed in the mouth and the ground electrode in the tail. Electrical signals generated in the retina were amplified (x1000) and filtered (band pass from 1 Hz to 1000 Hz) by a commercial amplifier (Digitimer Ltd, Letchworth Garden City, UK). The recorded signals were digitized (Power Lab; ADInstruments Pty. Ltd., Chalgrove, UK) and displayed on a PC computer. Bilateral ERG recording was performed simultaneously from both eyes. Light stimuli were calibrated before each experiment, and the calibration protocol was set to assure the same recording parameters for both eyes. The ERG responses were recorded by stimulating the retina with light intensities ranging between10^−6^ and 10^−4^ cd·s·m^−2^ for the scotopic threshold response (STR), 10^−4^ and 10^−2^ cd·s·m^−2^ for the rod response, and 10^−2^ and 10^2^ cd·s·m^−2^ for the mixed (rod and cone) response. For each light intensity level, a series of ERG responses were averaged (from 40 ERG responses for the dimmest stimulus intensities to 5 for the brightest stimulus), and the interval between light flashes was adjusted to ensure a timing that allowed response recovery (from 5 s for the dimmest stimulus intensities to 60 s for the brightest stimulus). At the end of each session the animals were treated with topical tobramicine (Tobrex®; Alcon-Cusí, S.A.) in both eyes. The analysis of the different recordings was performed with the normalization criteria established for the International Society for Clinical Electrophysiology of Vision (ISCEV) for the measures of the amplitude and implicit time of the different waves studied.

### Analysis and statistics

The STR was analyzed for each stimulus; pSTR was measured from baseline to the “hill” of the positive deflection, approximately (*ca.*) 110 ms from the flash onset; and nSTR was measured from baseline to first “valley” after pSTR, *ca.* 220 ms from the flash onset. The a-wave was measured from the baseline to the first valley, *ca.* 10 ms, from the flash onset; the b-wave amplitude was measured from the bottom of the a-wave valley to the top of the hill of the positive deflection. The time point of the b-wave measurement varied depending upon the intensity used. The implicit time was measured from the presentation of the stimulus to the top of the b-wave. Data from operated and unoperated eyes were compared; ERG wave amplitudes and implicit times were calculated for each animal group and the percentage difference between the operated and the unoperated eyes was obtained for each stimulus and was further averaged (mean±SEM). The results were analyzed with SigmaStat® 3.1 for Windows® (Systat Software, Inc., Richmond, CA). Descriptive statistics were calculated, the normality of the distribution of the data was examined with a normality test, and parametric or non-parametric test were used accordingly; *t*-test was used for the comparison between the absolute response of both eyes prior and post ONT and for the comparison of the percentage response of the operated eye versus the unoperated eye prior and post ONT. As an attempt to estimate a possible inter-relation between the progressions of response along studied times, an analysis of variance (ANOVA) on ranks test was used to compare the percentage response between different animal groups. The statistic significance was placed in a p<0.05 for all tests, and the statistic was always of two tails.

## Results

### Electroretinograms in control albino and pigmented rats

**Figure 1:**
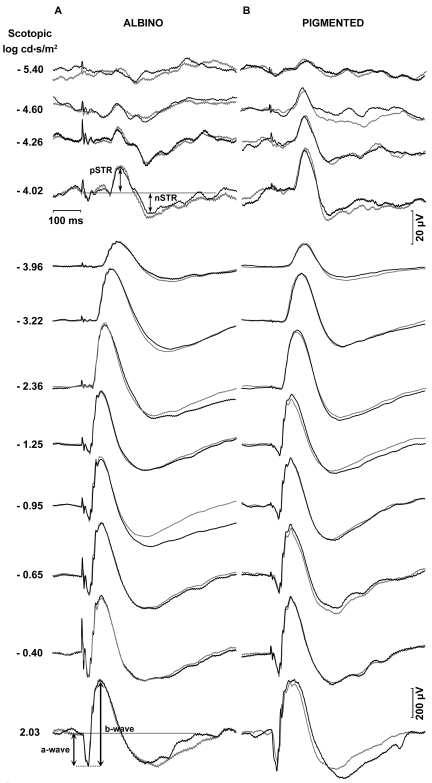
Scotopic electroretinographic recordings in albino and pigmented rats. Examples of the ERG traces recorded in an albino (**A**) and pigmented (**B**) rats in response to flash stimuli of increasing intensity. Thin traces correspond to recordings obtained from the right eye and bold traces correspond to recordings obtained from the left eye. The intensity of the flash stimuli is indicated in log cd·s/m^2^ units at the left of the recording traces. The scotopic threshold responses were elicited by weak light stimuli from −4.60 to −5.40 log cd·s/m^2^. Rod and mixed responses were elicited by light intensities from −3.96 to 2.03 log cd·s/m^2^. No significant difference in the ERG amplitudes between left and right eyes was observed in any of the studied animals. Examples for the measurement of wave amplitudes are shown in the albino rat recordings.

**Figure 2:**
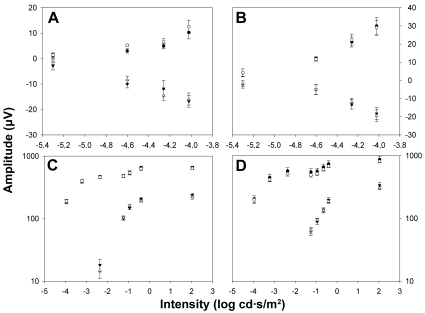
Electroretinographic amplitude measurements in albino and pigmented rats. Averaged data (mean±SEM) of ERG amplitudes versus stimulus intensities both from control albino (**A**, **C**; n=6) and pigmented rats (**B**, **D**; n=8) is shown for the right eye (open symbols) and left eye (filled symbols). **A, B**: Positive scotopic threshold response (circles) and negative scotopic threshold responses (triangles). **C**, **D**: Triangles correspond to the a-wave amplitude and circles correspond to the b-wave amplitudes. When the amplitudes obtained from the albino group were compared to those of the pigmented, there were no significant differences (*t*-test; p>0.05) for any of the waves analyzed, except the pSTR (*t*-test; p<0.05).

To study the effect of ONT on ERG waves in albino and pigmented rats, and as baseline measurements, simultaneous ERG recordings were taken of the right and left eyes of each animal before surgery. Figure 1 shows a representative example of the ERG traces recorded in an albino (Figure 1A) and pigmented (Figure 1B) rat in response to flash stimuli of increasing intensity. The STR were elicited by weak light stimuli (−5.4 to −4.02 log cd·s·m^−2^). The amplitudes of pSTR and nSTR increased exponentially with the intensity of the light stimulus both in albino and pigmented rats (Figure 2A,B). No ERG a-wave was observed for light intensities below −2.36 log cd·s·m^−2^ (Figure 1). The b-wave elicited by light intensities from −3.96 cd·s·m^−2^ increased exponentially, reaching its maximum for 2.03 cd·s·m^−2^ (Figure 1). Figures 2C,D show averaged data of ERG a- and b-wave amplitudes in albino and pigmented rats, respectively. No significant differences in any of the above ERG amplitudes between left and right eyes were observed in any of the animals of this study before surgery. When the amplitudes obtained from the albino group were compared to those of the pigmented, there were no significant differences (*t*-test; p>0.05) for any of the waves analyzed except for the pSTR (*t*-test; p<0.05), an unexpected result for which, at present, we have no clear explanation.

### Electroretinograms in experimental albino and pigmented rats with left ONT

ERG recordings were taken simultaneously from the right unoperated eye and the left operated eye in albino and pigmented rats at increasing survival intervals after ONT.

### One week after ONT

ERG traces from albino animals in group I (n=6) examined one week after ONT showed reductions in the pSTR and nSTR responses of approximately 40% when compared to the unoperated contralateral eye (*t*-test, p<0.001). At this time, scotopic and mixed ERG recordings of the operated eye also showed reduced a- and b-wave amplitudes when compared with the unoperated eye. Moreover, the ERG recordings from pigmented animals in group V (n=8), examined one week after ONT, showed mainly a decrease in the pSTR response of approximately 40% (*t*-test, p<0.001). At this time, the remainder of the responses recorded in the operated eye also show reduced amplitudes when compared with the unoperated eye.

### Two weeks after ONT

**Figure 3:**
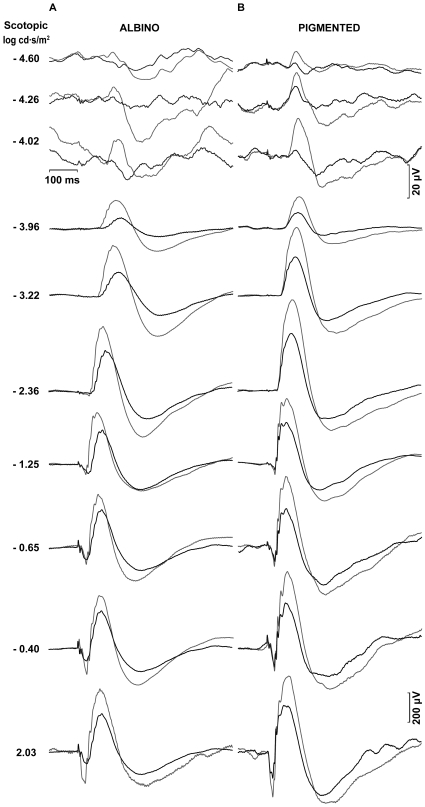
Scotopic electroretinographic recordings two weeks after optic nerve transection. Examples of the ERG traces recorded in an albino (**A**) and pigmented (**B**) rat in response to flash stimuli of increasing intensity for the unoperated right eye (thin traces) and for the operated left eye (bold traces) two weeks after optic nerve transection. The intensity of the flash stimuli is indicated to the left of the recording traces. Reduction in the pSTR and nSTR responses from operated eyes versus unoperated eyes is clearly evident in both albino and pigmented rats.

**Figure 4:**
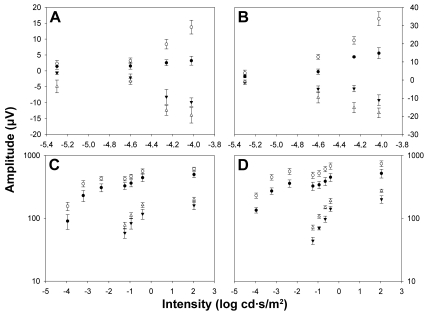
Electroretinographic amplitude measurements two weeks after optic nerve transection. Averaged data (mean±SEM) of ERG amplitudes versus stimulus intensity both from albino (**A**, **C**; n=7) and pigmented rats (**B**, **D**; n=5) from recordings obtained two weeks after ONT. Open symbols show data averaged from unoperated right eyes and filled symbols show data averaged from operated left eyes. **A**, **B**: Data corresponding to positive scotopic threshold responses (pSTR) are shown as circles and that corresponding to negative scotopic threshold responses (nSTR) are shown as triangles. **C**, **D**: Amplitudes corresponding to the a-wave are shown as triangles and those corresponding to b-wave are shown as circles. A significant reduction in the wave amplitudes of the pSTR, nSTR, b-wave scotopic response, and a- and b-wave was observed in the operated eyes (*t*-test; p<0.001) when compared to the unoperated control eyes.

Representative ERG traces from an albino rat in group II (n=7) recorded from operated (bold trace) and unoperated (thin trace) eyes two weeks after left ONT are shown in Figure 3A. Reduced pSTR and nSTR responses from operated eyes amounted to 65% of those of the unoperated eyes (*t*-test, p<0.001), and these are clearly observed at two weeks after ONT (Figure 3A). At this time, scotopic and mixed ERG recorded in operated eyes also showed reduced a- and b-wave amplitudes when compared with the unoperated eye (*t*-test, p<0.001). Averaged data of ERG wave amplitudes from animals in group II are shown in Figures 4A,C.

Figure 3B shows ERG traces from a representative pigmented rat in group VI (n=5) recorded from operated (bold trace) and unoperated (thin trace) eyes two weeks after the ONT. Reduced pSTR and nSTR responses from operated eyes amounted to 55% of those of the unoperated eyes (*t*-test, p<0.001). At this time, scotopic and mixed ERG recorded in operated eyes also showed reduced a- and b-wave amplitudes when compared with unoperated eyes (*t*-test, p<0.001). Averaged data of ERG wave amplitudes from animals in group VI are shown in Figures 4B,D.

### Four weeks after ONT

**Figure 5:**
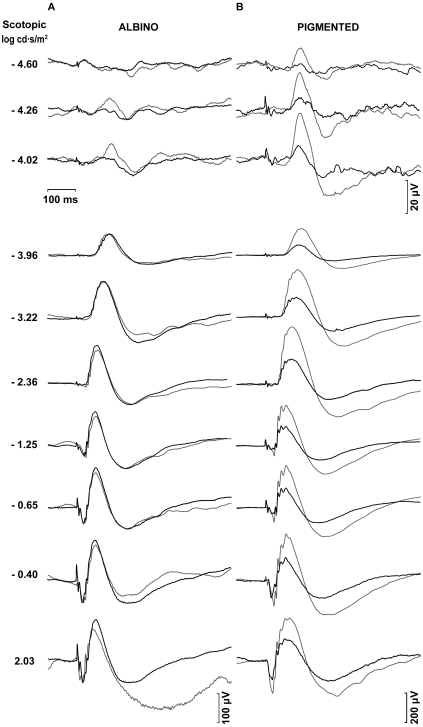
Scotopic electroretinographic recordings four weeks after optic nerve transection. Examples of the ERG traces recorded in an albino (**A**) and pigmented (**B**) rats in response to flash stimuli of increasing intensity four weeks after optic nerve transaction. Thin traces correspond to the unoperated right eye and bold traces correspond to the operated left eye. The intensity of the flash stimuli is indicated to the left of the recording traces. Reduced pSTR and nSTR responses from operated eyes versus control eyes were clearly observed for both strains, while an apparent recovery of the ERG wave amplitudes was observed for the scotopic and mixed responses in the albino rats.

**Figure 6:**
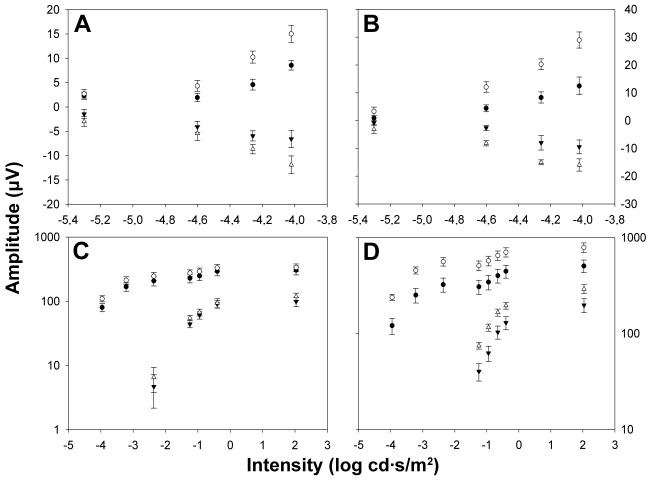
Electroretinographic amplitude measurements four weeks after optic nerve transection. Averaged data (mean±SEM) of ERG amplitudes versus stimulus intensity both from albino (**A**, **C**; n=9) and pigmented rats (**B**, **D**; n=6) is shown from recordings obtained four weeks after ONT. Open symbols show data averaged from unoperated right eyes and filled symbols show data averaged from operated left eyes. **A**, **B**: Data corresponding to positive scotopic threshold responses (pSTR) are shown as circles and that corresponding to negative scotopic threshold responses (nSTR) are shown as triangles. **C**, **D**: Amplitudes corresponding to the a-wave are shown as triangles and those corresponding to b-wave are shown as circles. A significant reduction in the wave amplitudes of the pSTR, nSTR, b-wave scotopic response, and a- and b-wave was observed in the operated eyes (*t*-test; p<0.001) when compared with the unoperated eyes. The significant reduction of ERG wave amplitudes in operated eyes versus unoperated eyes is evident only in the pSTR and nSTR responses (p<0.001) in albino rats. A significant reduction for the pSTR, nSTR, b-wave scotopic response, and a- and b-wave of the mixed response was observed in pigmented rats (p<0.001).

Four weeks after ONT, differences in ERG wave amplitudes between operated and unoperated eyes were also observed in group III (n=9) albino rats. Figure 5A shows the ERG traces from a single representative animal of group III illustrating the reductions in the pSTR and nSTR responses of approximately 40% in the operated eyes versus unoperated eyes (*t*-test, p<0.001). However, at this time, there was an apparent recovery of the ERG wave amplitudes observed for the scotopic and mixed responses. Averaged data of ERG wave amplitudes from animals in group III are shown in Figures 6A, C.

Four weeks after ONT, differences between ERG wave amplitudes of operated and unoperated eyes were also observed in group VII (n=6) pigmented rats. Figure 5B shows the ERG traces from a single representative animal of group VII illustrating the reductions in the pSTR and nSTR responses of approximately 60% in the operated eyes versus unoperated eyes (*t*-test, p<0.001). At this time, scotopic and mixed ERG recorded in operated eyes also show reduced a- and b-wave amplitudes when compared with unoperated eyes (*t*-test, p<0.001). Averaged data of ERG wave amplitudes from animals in group VII are shown in Figures 6B,D.

### Twelve weeks after ONT

**Figure 7:**
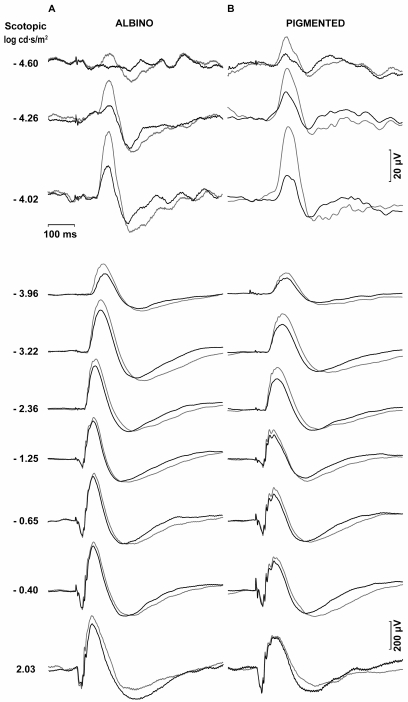
Scotopic electroretinographic recordings 12 weeks after optic nerve transection. Examples of the ERG traces recorded in an albino (**A**) and pigmented (**B**) rats in response to flash stimuli of increasing intensity for the unoperated right eye (thin traces) and for the operated left eye (bold traces) 12 weeks after optic nerve transection. The intensity of the flash stimuli is indicated to the left of the recording traces. Reduction in the pSTR and nSTR responses from operated eyes versus unoperated eyes was clearly seen 12 weeks after ONT, while no other apparent difference in ERG scotopic and mixed responses was evident between the operated and unoperated animals for both rat strains.

**Figure 8:**
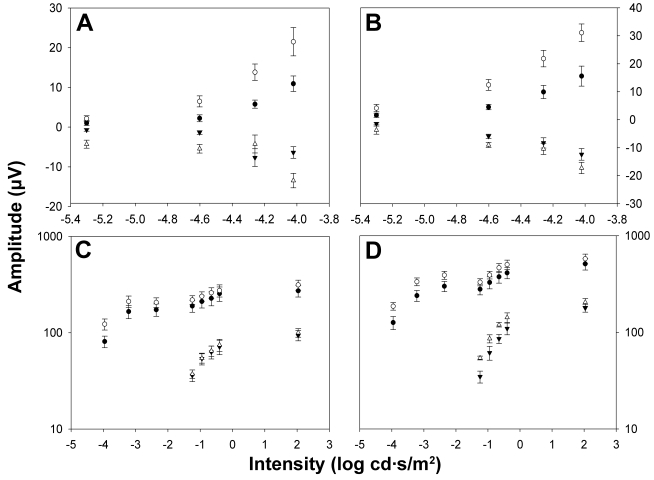
Electroretinographic amplitude measurements 12 weeks after optic nerve transection. Averaged data (mean±SEM) of ERG amplitudes versus stimulus intensity both from albino (**A**, **C**; n=9) and pigmented rats (**B**, **D**; n=5) from recordings obtained twelve weeks after ONT. Open symbols show data averaged from unoperated right eyes and filled symbols show data averaged from operated left eyes. **A**, **B**: Data corresponding to positive scotopic threshold responses (pSTR) are shown as circles and that corresponding to negative scotopic threshold responses (nSTR) are shown as triangles. **C**, **D**: Amplitudes corresponding to the a-wave are shown as triangles and those corresponding to b-wave are shown as circles. A significant reduction of ERG wave amplitudes in operated eyes versus unoperated eyes is observed for the pSTR and nSTR responses (p<0.001) in both strains of rat. There was a small reduction in the b-wave scotopic response and a- and b-wave of the mixed response in pigmented rats (p<0.001).

Twelve weeks after ONT, changes in ERG wave amplitudes were still observed in operated eyes from group IV rats (n=9; Figure 7A). At this point after ONT, the reduced pSTR and nSTR responses from operated eyes were approximately 60% of those values recorded for the unoperated eyes (*t*-test, p<0.001), while no other apparent differences in ERG scotopic and mixed responses were observed between operated and unoperated eyes. Averaged data of ERG wave amplitudes from animals in group IV are shown in Figures 8A,C.

In pigmented rats, twelve weeks after the ONT, changes in ERG wave amplitudes were still observed in operated eyes from group VIII (n=5; Figure 7B). At this point after ONT, the reduced pSTR and nSTR responses from operated eyes amounted to approximately 55% of those values recorded for the unoperated eyes (*t*-test, p<0.001), while no other apparent differences in ERG scotopic and mixed responses were observed between operated and unoperated eyes. Averaged data of ERG wave amplitudes from animals in group VIII are shown in Figures 8B,D.

**Figure 9:**
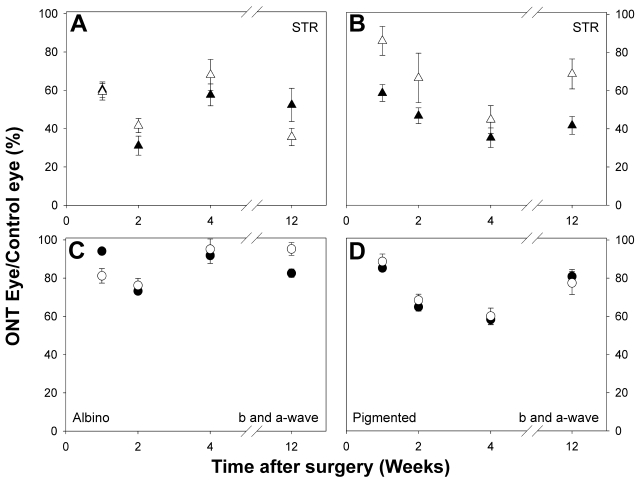
Evolution of electroretinographic wave amplitudes over a 12-week time period. Average data (mean±SEM) of the reduction in the ERG wave amplitudes is represented as the percentage between operated and unoperated eyes for 1, 2, 4, and 12 weeks after ONT, both from albino (**A**, **C**) and pigmented rats (**B**, **D**). **A**, **B**: Close triangles show averaged amplitudes for the positive scotopic threshold response (pSTR) and open triangles show averaged amplitudes for the negative scotopic threshold responses (nSTR). **C**, **D**: Open circles show averaged data for the a-wave amplitudes and closed circles show averaged data for the b-wave amplitude. A permanent reduction in the pSTR and nSTR was observed for the 12-week time period both for pigmented and albino rats. The reduction in the a-wave and b-wave amplitudes recovered earlier in albino rats than in pigmented rats.

Our data suggest that reductions in the pSTR and nSTR continue a long time after ONT, and thus appear to be permanent (Figures 9A,B) for both albino and pigmented rats. However, the changes observed in the a- and b-wave amplitudes of the scotopic and mixed ERG are obvious at a short time (1–4 weeks) but not a long time after surgery (12 weeks). Moreover, a comparison between data from albino and pigmented rats show that the reductions in the scotopic and mixed ERG responses observed at short intervals (1–4 weeks) recovered earlier in albinos than in pigmented rats (Figure 9C,D).

## Discussion

Previous studies by our group have quantified the time course and amount of RGC loss induced by ONT close to the eye [27,28,30–32]. Here we have further extended our previous work and investigated the relationship between RGCs and the components of the full-field electroretinogram by performing ONT and comparing the responses obtained in albino and pigmented rats at different survival intervals ranging 1 to 12 weeks. Our results document that ONT induces transient alterations of the major ERG components, the a- and b-wave; and provokes permanent diminutions of the early components of the ERG, the negative and positive scotopic threshold responses. Overall, our results are in agreement with previous studies of pigmented rats [7,44], and provide new information regarding the adult albino rat.

The need to understand and diagnose eye diseases in which RGCs are affected has awakened a large interest in developing techniques to assess these cells functionally. The flash ERG registers global retinal electrical activity after a light stimulus and is used mostly to assess the function of several neural populations of the retina, e.g., the photoreceptors and bipolars, which are associated with the a- and b-waves, the main waves of the ERG. In addition, recording of scotopic threshold responses elicited with light intensities several orders of magnitude smaller than those needed to elicit the b-wave appears to be a fine and sensitive functional test for the RGC population [4–9,11]. Indeed, in adult pigmented rats, Naarendorp and colleagues (2001) [52] injected 100 µM NMDA (N-Methyl-d-Aspartate) intraocularly to eliminate contributions by the inner retina to the ERG response elicited with dim lights, and found suppression of the STR recordings but not of the b-wave, thus providing additional evidence for the inner retinal origin of these potentials. Moreover, intraocular injection of tetrodotoxin (TTX) in rats, or GABA in mice, induced significant reductions in the STR amplitudes elicited with dim lights, both in pigmented rats [7] and mice [9], providing further evidence for the inner retinal origin of these recordings.

In our analysis of the functional changes of the albino retina evolving from 1 to 12 weeks after ONT, we found a decrease in both STR waves of approximately 40% at 1 week and approximately 65% at 2 weeks after ONT. Moreover, in both strains (pigmented and albino) the effects of the ONT on the STR waves were comparable, that is, in both strains there was an significant reduction of the STR waves—a conclusion that is obtained from data analysis in which the values registered from the operated eyes was compared with the unoperated control eyes. The pSTR and nSTR responses at 12 weeks, the longest time examined, were comparable in the pigmented and the albino rats (Figure 9). The decrease recorded in STRs, which was first evident by 1–2 weeks after ONT, coincides in time with the major wave of RGC loss that follows ONT in the adult rat; indeed, approximately 80% of the RGC population is lost during the first two weeks [27,28,30,31,53]. A similar decrease in amplitude for STR waves has been reported following optic nerve transection in the adult pigmented rat [7]. In our experiments, the decrease in STR wave amplitude was permanent, because 12 weeks after ONT, the last time point examined in the present study, the STR responses were still reduced, at approximately 40% of the normal value, both in albino and pigmented rats. At this time after ONT, the population of RGCs surviving in the retina is approximately 5%–10% of the original RGC population [28,54], thus it is conceivable that the main reduction of approximately 50%–60% of the STR responses is due to the loss of RGCs, the main neuronal population affected by ONT. Indeed, previous work has suggested that ONT does not affect the survival of non-RGC neurons [28,42,55], although the possibility of retrograde transneuronal degeneration of first and second order neurons in the retina following ONT cannot be completely discounted [56–58]. We didn’t look at how much of the 40% amplitude of the STR could be elicited by the residual population of RGCs surviving in the adult rat retina at 12 weeks, and it is plausible that the population of amacrine cells, which are not affected by ONT, also contribute to this wave, as previously shown in other species (e.g., mice, cats, and humans; 7, 10) as well as in transgenic mice lacking RGCs [59]. Thus, overall, the progressive diminution in the amplitude of the STR waves observed shortly after ONT, and its persistence 12 weeks later, highlights the importance of the STR recordings as an electrophysiological tool for the assessment of RGC function in these laboratory animals.

One week after ONT there was a significant reduction in the a- and b-waves, these reductions were still present two weeks after ONT in albino and pigmented rats, but had recovered to almost basal levels by 4 weeks in the albinos and by 12 weeks in the pigmented rats. A similar trend for diminution and recovery of the a- and b-waves was found in pigmented rats with their ON transected [7,43], in which the a- and b-waves were reduced to approximately 80%–85% of their normal amplitude when compared to the unoperated eye. The release of trophic factors by macro and microglia activation in these injured retinas [33] may play a role in the amplitude of these waves as previously suggested [43], but it is also possible that transient down-regulation of photoreceptor-specific genes after ONT could explain the reduction of the major ERG components. Indeed, recent work from this laboratory has shown that several genes whose transcription products are involved in phototransduction, such as rhodopsin, opsin, or recoverin, are transiently down-regulated [40] as soon as 12 h after ONT, when gene transcription is severely halted and mRNA levels diminish to approximately one half of their normal values. Interestingly, the basal values of these mRNAs recover slowly within the next weeks [39].

The adult rat is widely used in a range of experimental models for several neurologic diseases involving RGC injury, and several morphological techniques are available to quantify the effects of these injuries on the survival and rescue of rat RGCs ex vivo [26–30,49,60] and in vivo [61]. However, there are few functional techniques to assess the RGC population in vivo, including the multifocal ERG [62] or the pattern ERG [63], thus highlighting the importance of the STR recordings to identify this population of neurons in the adult albino or pigmented rat retina. Our present studies on albino and pigmented rats, as well as those of others on pigmented rats [7], underline the potential use of the STR components of the ERG as a functional index to demonstrate in vivo RGC dysfunction in several experimental models involving RGC injury, such as an acute [64,65] or chronic increase in intraocular pressure [66–68].

In summary, we examined the alterations induced in the STR components of the ERG and found a clear and persistent diminution of these components over time following ONT in the albino and pigmented rat. This functional approach could be of great interest for several animal models covering degeneration of the RGC population, for which, to date, there have been few functional tests.
